# Epidemiology of Candidemia in Mashhad, Northeast Iran: A Prospective Multicenter Study (2019–2021)

**DOI:** 10.3390/jof10070481

**Published:** 2024-07-12

**Authors:** Somayeh Dolatabadi, Mohammad Javad Najafzadeh, Abbas Raeisabadi, Hossein Zarrinfar, Mahsa Jalali, Bram Spruijtenburg, Eelco F. J. Meijer, Jacques F. Meis, Cornelia Lass-Flörl, Theun de Groot

**Affiliations:** 1 Department of Biology, Hakim Sabzevari University, Sabzevar 9617976487, Iran; 2 Department of Medical Parasitology and Mycology, Faculty of Medicine, Mashhad University of Medical Sciences, Mashhad 91766-99199, Iran; 3 Department of Medical Mycology, Faculty of Medicine, Mazandaran University of Medical Sciences, Sari 48471-91628, Iran; 4 Allergy Research Center, Mashhad University of Medical Sciences, Mashhad 91766-99199, Iran; 5 Radboudumc-CWZ Center of Expertise for Mycology, 6532 SZ Nijmegen, The Netherlandseelco.meijer@cwz.nl (E.F.J.M.);; 6 Canisius-Wilhelmina Hospital (CWZ)/Dicoon, 6532 SZ Nijmegen, The Netherlands; 7 Institute of Translational Research, Cologne Excellence Cluster on Cellular Stress Response in Aging-Associated Diseases (CECAD), Excellence Center for Medical Mycology (ECMM), University of Cologne, 50931 Cologne, Germany; 8 Institute of Hygiene and Medical Microbiology, Medical University of Innsbruck, Excellence Center for Medical Mycology (ECMM), 6020 Innsbruck, Austria

**Keywords:** candidemia, *Candida albicans*, antifungal resistance, genotyping, short tandem repeats, *Candida parapsilosis*

## Abstract

Candidemia is a major cause of morbidity and mortality in health care settings, and its epidemiology is changing. In the last two decades, the proportion of non-*albicans Candida* (NAC) yeasts in candidemia has increased. These yeasts more often display resistance to common antifungals. In many western countries, candidemia is mainly caused by susceptible *C. albicans*, while in resource-limited countries, including Iran, the candidemia species distribution is studied less often. Here, we investigated the species distribution, resistance levels, and characteristics of patients with candidemia in five hospitals in Mashhad (northeast Iran) for two years (2019–2021). Yeast isolates from blood were identified with MALDI-TOF MS and subjected to antifungal susceptibility testing (AFST) using the broth microdilution method, while molecular genotyping was applied to *Candida parapsilosis* isolates. In total, 160 yeast isolates were recovered from 160 patients, of which the majority were adults (60%). Candidemia was almost equally detected in men (48%) and women (52%). Almost half of patients (*n* = 67, 49%) were from intensive care units (ICUs). *C. parapsilosis* (*n* = 58, 36%) was the most common causative agent, surpassing *C. albicans* (*n* = 52, 33%). The all-cause mortality rate was 53%, with *C. albicans* candidemia displaying the lowest mortality with 39%, in contrast to a mortality rate of 59% for NAC candidemia. With microbroth AFST, nearly all tested isolates were found to be susceptible, except for one *C. albicans* isolate that was resistant to anidulafungin. By applying short tandem repeat (STR) genotyping to *C. parapsilosis,* multiple clusters were found. To summarize, candidemia in Mashhad, Iran, from 2019 to 2021, is characterized by common yeast species, in particular *C. parapsilosis*, for which STR typing indicates potential nosocomial transmission. The overall mortality is high, while resistance rates were found to be low, suggesting that the high mortality is linked to limited diagnostic options and insufficient medical care, including the restricted use of echinocandins as the first treatment option.

## 1. Introduction

Candidemia is a growing concern in hospital settings, posing every year a serious health threat to hundreds of thousands of patients worldwide [1,2]. It is one of the most common bloodstream infections, both in adult and pediatric patients. The species *Candida albicans*, *C*. *glabrata* (also known as *Nakaseomyces glabrata*), *C*. *parapsilosis*, *C*. *tropicalis*, and *C*. *krusei* (also known as *Pichia kudriavzevii*) are the five leading causative agents of candidemia, accounting for approximately 85–90% of candidemia [1,3]. However, these five most common species display notable regional differences. *C*. *albicans* is the most common etiological agent of candidemia in the United States and most European countries, although its proportion as compared to other *Candida* species is decreasing in the last decade [4,5,6]. Among these five common species, *C*. *albicans*, together with *C. tropicalis*, is regarded as the most virulent species, while it shows the lowest rate of antifungal resistance [1,7,8,9]. *C. glabrata* is the second most common cause of candidemia in the USA and many North and West European countries [4,5,10]. Moreover, it is the first cause of candidemia in intensive care units and in patients with hematological malignancies and solid tumors [11]. The third most common yeast species is *C. parapsilosis*, especially prevalent in South European countries [12]. This yeast can easily spread through the hands of healthcare workers, and azole resistance is frequently observed. Additionally, *C. parapsilosis* has shown prolonged survival within hospital wards and can be the source of clonal outbreaks [13]. Another common *Candida* species is *C. tropicalis*, which is associated with the highest mortality rates among *Candida* species and is the first or second cause of candidemia in developing countries, such as India and Brazil, with resistance steadily increasing [9,14,15]. In Iran, the majority of studies report *C. albicans* as most common in candidemia, although other yeasts are emerging, as well as antifungal resistance [16,17,18].

Depending on the species, the mortality rate may vary from approximately 30 to 70% [1]. The increase in mortality is associated with an increase in non-*albicans Candida* (NAC) yeast species. A likely explanation is resistance to the limited number of antifungal drugs available to treat candidemia in developing countries [6]. Especially the reduced susceptibility of the NAC yeast species *C. glabrata, C. tropicalis*, and *C. parapsilosis* to fluconazole, the most inexpensive and readily available antifungal agent used to treat candidemia, is highly problematic in these countries [19]. Moreover, azole-resistant *C. parapsilosis* often spread clonally and are persistent within healthcare environments [20]. This problem is further aggravated by the limited use of accurate identification techniques in Iran, such as sequencing or matrix-assisted laser desorption ionization time-of-flight mass spectrometry (MALDI-TOF MS), which leads to an unselective application of antifungals, inducing the development and persistence of resistant species [1,6].

These issues emphasize the importance of conducting epidemiological studies to explore species distribution, the burden of antifungal resistance, and to characterize the clinical profile of patients with candidemia. Considering the limited number of studies on candidemia in Iran, we performed a two-year retrospective candidemia study in Mashhad, Iran, in which we collected clinical data of patients, assessed species distribution by MALDI-TOF, and determined their antifungal susceptibility pattern, along with short tandem repeat (STR) genotyping of *C. parapsilosis*, to improve our insights on candidemia.

## 2. Materials and Methods

### 2.1. Study Design and Sample Processing

This study retrospectively included patients with candidemia admitted to five hospitals in Mashhad, Iran, including Ghaem (800 beds), Emam Reza (948 beds), 22 Bahman (175 beds), Arya (100 beds), and Dr. Sheikh (150 beds), during July 2019 to July 2021. All centers were multi-specialty hospitals, except for Dr. Sheikh, which is a pediatric hospital. Blood samples were inoculated in Bactec 9120 blood culture bottles (Becton Dickinson, Spark, MD, USA). From positive bottles, 150 µL was streaked on Sabouraud dextrose agar (SDA, Merck, Darmstadt, Germany) and CHROMagar Candida (CHROMagar, Paris, France) plates, which were subsequently incubated at 37 °C for 24–48 h. Colony morphology (color, shape, and size) was visually inspected to identify samples potentially harboring more than one species. Colonies with different morphologies were transferred to SDA plates, incubated at 37 °C for 24–48 h, and subjected to further analyses. All yeast isolates were identified using a microflex LT MALDI-TOF MS system (Bruker Daltonics, Bremen, Germany) and a full extraction method according to manufacturer instructions [21]. Candidemia or invasive candidiasis was diagnosed when blood cultures yielded yeast. Mortality was reported as the all-cause mortality rate during study period. Patients < 16 years were considered children and ≥16 years adults. Therapeutic failure according to expert opinion was defined as persistent positive blood cultures (with yeasts) despite antifungal treatment for 7 days. This study was approved by the ethic committee of Mashhad University of Medical Sciences (ethical approval number IR.NIMAD.REC.1398.103).

### 2.2. In Vitro Antifungal Susceptibility Testing (AFST)

AFST was performed using the broth microdilution method according to the Clinical and Laboratory Standards Institute (CLSI) protocol (CLSI M27, 4th editon) [22]. The following antifungal drugs were tested: fluconazole (FLU), amphotericin B (AMB) (both from Sigma Chemical Corporation, St. Louis, MO, USA), voriconazole (VOR; Pfizer, New York, NY, USA), micafungin (MFG; Astellas Pharma, Ibaraki, Japan), and anidulafungin (AFG, Pfizer, New York, NY, USA). Plates were incubated at 37 °C for 24 h and minimum inhibitory concentrations (MICs) were recorded after visual examination. Reference strains of *C*. *parapsilosis* (ATCC 22019) and *C*. *krusei* (ATCC 6258) were used for quality control. The MIC data were categorized according to clinical breakpoints. Isolates were classified as susceptible (S), susceptible dose-dependent (SDD) or intermediate (I), and resistant (R). If clinical breakpoints were not available, MICs were interpreted according to epidemiological cut-off values (ECVs) and isolates were classified as wild-type (WT) at MIC ≤ ECV or non-wild type (NWT) at MIC > ECV, according to the CLSI M57 document [23]. 

### 2.3. Multiplex Short Tandem Repeat (STR) Genotyping

*C. parapsilosis* DNA was extracted and purified with the MagNA Pure and Viral NA Small volume kit, and the MagNA Pure 96 instrument (All Roche Diagnostics GmbH, Mannheim, Germany), as previously described [23]. STR multiplex PCR genotyping was performed on a thermocycler (Biometra, Göttingen, Germany) using 1× FastStart Taq polymerase buffer without MgCl2, deoxynucleotide triphosphates (dNTPs) (0.2 mM), MgCl_2_ (3 mM), forward and reverse primers (10 µM), 1 U FastStart Taq polymerase (Roche Diagnostics), and isolated DNA [24]. STRs were amplified with a thermal protocol of denaturation at 95 °C for 10 min, followed by 30 cycles consisting of annealing at 60 °C for 30 s, extension at 72 °C for 1 min, and a final incubation for 10 min at 72 °C. Amplicons were diluted 1:200 in water and 10 µL of diluted amplicon in addition to 0.12 µL of Orange 500 DNA size standard (Nimagen, Nijmegen, The Netherlands) were incubated for 1 min at 95 °C and analyzed on a 3500 XL genetic analyzer (Applied Biosystems, Foster City, CA, USA). Copy numbers of STR markers were determined using the Genemapper 5 software (Applied Biosystems), and relatedness between isolates was analyzed as previously described [25].

## 3. Results

### 3.1. Patients’ Characteristics and Species Distribution

A total of 160 yeast isolates were collected during two years from 160 patients of whom, a majority were adults (*n* = 96, 60%) (Table 1). Candidemia was almost equally detected in men (*n* = 83, 52%) and women (*n* = 77, 48%), and almost half of the yeast isolates were recovered from ICUs (*n* = 79, 49%). Using MALDI-TOF MS identification, *C. parapsilosis sensu stricto* (*n* = 58, 36%) was the most common causative agent, followed by *C. albicans* (*n* = 52, 33%), while no coinfections were observed. For 156 patients, outcomes were known, showing an all-cause mortality rate of 53%. *C. albicans* candidemia coincided with a relative low mortality rate of 39% as compared to NAC candidemia, which had a mortality rate of 59%. In the current study, the most commonly used antifungals included fluconazole (*n* = 48, 30%) and liposomal amphotericin B (*n* = 40, 25%). More than one antifungal drug was administered to 12 patients (8%), while 54 patients (34%) received no antifungal therapy. Remarkably, patients with *C. albicans* were virtually all treated with an antifungal, while most patients with *C. parapsilosis* were not treated.

### 3.2. Resistance Investigation

In vitro AFST was performed on 131 *Candida* isolates according to the CLSI M27 guidelines. Overall, the tested antifungals demonstrated potent activity with only few cases of resistance (Table 2). For fluconazole, one *C. lusitaniae* isolate was NWT, while for amphotericin B, one NWT *C. albicans* isolate was found, according to CLSI M59 ECVs (Table S1). For echinocandins only a few *C. albicans* isolates showed elevated MICs. For anidulafungin one isolate demonstrated a MIC of 1 µg/mL, while there were also three intermediate susceptible isolates. From the latter three, two isolates were also intermediate susceptible for micafungin (Table S1). The less echinocandin susceptible *C. albicans* were isolated from patients who were not treated with echinocandins.

### 3.3. C. parapsilosis STR Genotyping Shows Clusters

**Figure 1:**
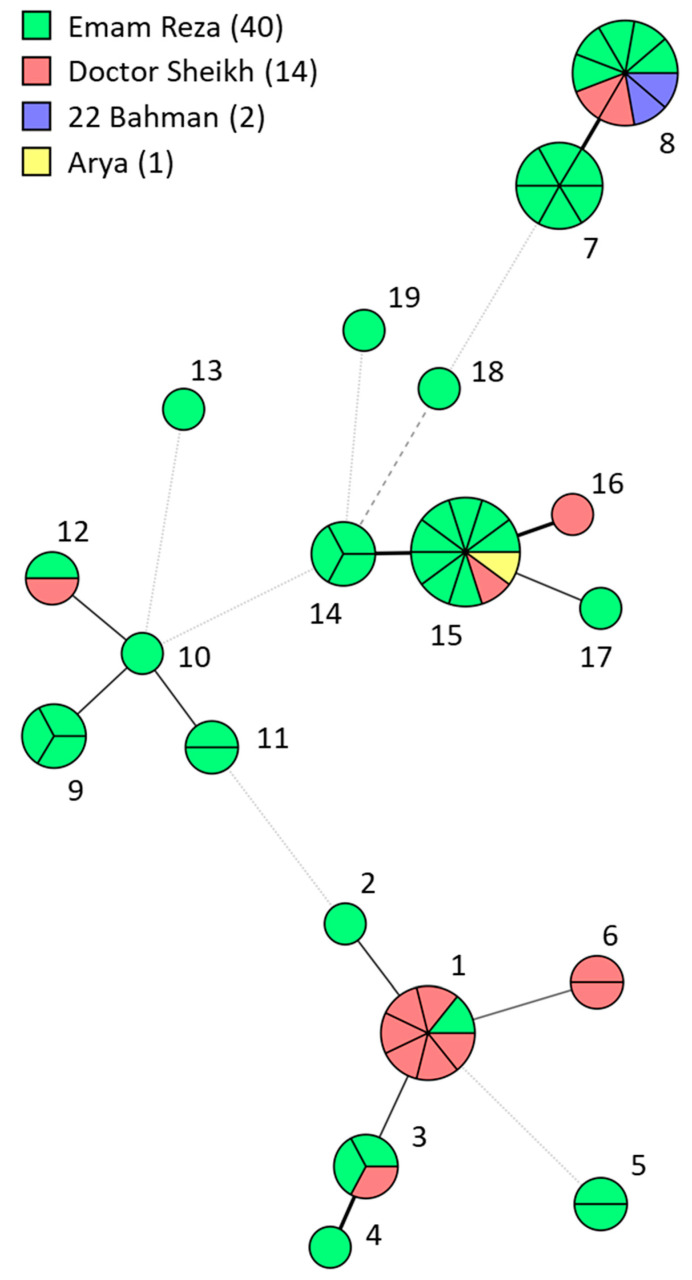
Minimum spanning tree of 57 *C. parapsilosis* isolates marked after the hospital of origin. Branch lengths indicate relatedness according to STR markers with thick solid lines (variation in one marker), thin solid lines (variation in two markers), thin dashed lines (variation in three alleles) and thin dotted lines (variation in four or more markers). Isolates are colored after the hospital and the number of isolates per hospital is shown in the color key. Genotype numbers correspond with Figure S1.

**Figure 2:**
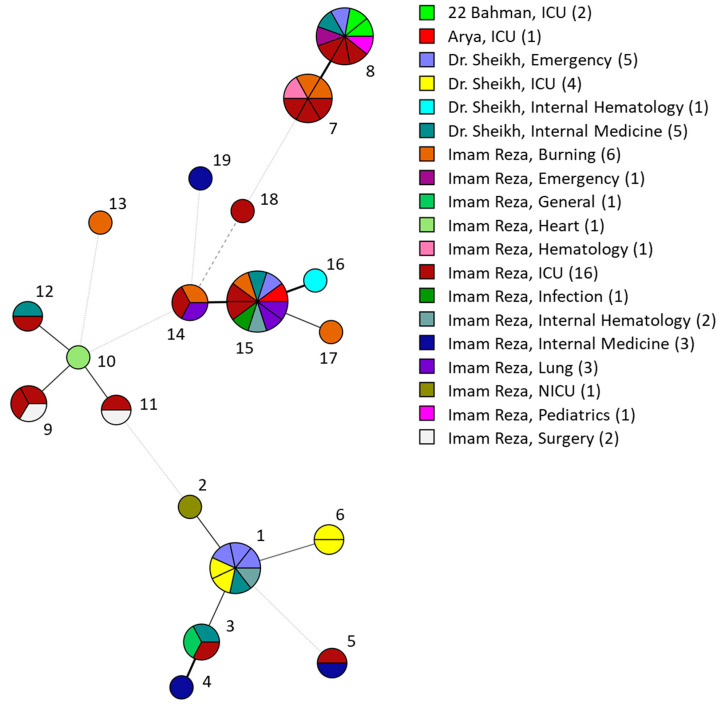
Minimum spanning tree of 57 *C. parapsilosis* isolates marked after the hospital with ward of origin. Branch lengths indicate relatedness according to STR markers with thick solid lines (variation in one marker), thin solid lines (variation in two markers), thin dashed lines (variation in three alleles) and thin dotted lines (variation in four or more markers). Isolates are colored after the hospital and ward and the number of isolates per hospital with ward is shown in the color key. Genotype numbers correspond with Figure S1. ICU, intensive care unit; NICU, neonatal intensive care unit.

To further investigate the high incidence of *C. parapsilosis* and the potential role of nosocomial transmission, we determined the phylogenetic relatedness between the 57 *C. parapsilosis* isolates by amplifying six microsatellite markers using multiplex PCR. The STR genotyping yielded 19 different genotypes, containing one to ten isolates (Figure S1). Isolates differed in four markers at most between each other. A total of seven clusters (≥3 isolates) were found, of which four spanned isolates from two or three hospitals (Figure 1). Many of these clusters were closely related to each other, like genotypes 7 to 8, and 14 to 16, differing only in the ploidy of one or two markers, respectively. Only the genotype 9 cluster was restricted to one hospital and not closely related (>2 markers difference) to any other cluster. Within all clusters, isolates originated from two to eight departments (Figure 2). For example, the largest cluster of ten isolates contained isolates from three hospitals, totaling eight different departments.

## 4. Discussion

### 4.1. Epidemiology

In the present study, we investigated the epidemiology of candidemia in five hospitals in Mashhad for a limited period of two years. The male-female ratio was overall comparable, which is in line with previous studies [6]. We found that *C. parapsilosis* (36%) was the leading candidemia agent, followed by *C. albicans* (33%). While the majority of Iranian studies identified *C. albicans* as most common species, a small nationwide study also found *C. parapsilosis* as most common [26,27]. Interestingly, some centers reported a *C. glabrata* frequency of 20–23% [28,29,30], which is higher than the 10% we found. Probably these differences in species distribution are to some extent due to different patient populations, as the average age of our patients was much lower. Previous epidemiological studies demonstrated that in elderly patients *C. glabrata* candidemia occurs more often, while pediatric or neonatal patients are more prone to *C. albicans* and *C. parapsilosis* candidemia episodes [3,6]. Nonetheless, the proportion of *C. albicans* in pediatric patients in our study (37%) was still much lower than the 57% found in the study from Kord et al., which was performed in neonatal and pediatric intensive care units in Tehran [29]. This difference might indicate a changing epidemiology, as the Tehran study was conducted between 2014 to 2016, while recent studies report more NAC yeasts [3]. 

Another factor influencing species distribution can be antifungal prophylaxis. Patients that underwent fluconazole prophylaxis are known to experience more *C. krusei* candidemia, as this species has intrinsic elevated MICs to this agent [31]. However, in the previously mentioned Iranian studies [27,28,29,30], the proportion of *C. krusei* is low, possibly attributable to the restricted use of antifungal prophylaxis. Additionally, whereas *C. tropicalis* is the most frequent species in some countries, the proportion in this and previous Iranian studies was relatively low (<15%) [27,28,29,30]. Among the other yeast species, we found *C. lusitaniae* and *Meyerozyma guilliermondii* (former *Candida guilliermondii*), which are known for intrinsic antifungal resistance, potentially limiting antifungal options that could result in treatment failure [32].

The overall mortality of 53% was a bit lower than worldwide estimations (64%), but higher than previous Iranian and European studies, which ranged from 28% to 48% [2,10,27,28,29,30]. We found that the mortality rate of *C. albicans* candidemia was lower when compared to other species, while *C. albicans* and *C. tropicalis* are considered as the *Candida* species with the highest virulence [6]. This is likely attributable to the antifungal treatment in our cohort, as most of patients with *C. albicans* were treated with one or more antifungals, while more than half of patients with *C. parapsilosis* and one third of patients with *C. tropicalis* was not treated with any antifungal. The high mortality rates of patients with *C. glabrata* and *C. krusei*, whom were also almost all treated with antifungals, might be explained by the usage of fluconazole despite both species are intrinsically resistant or display naturally elevated MICs for this drug [33]. These findings emphasize the importance of broad candidemia surveillance, rapid and accurate identification and adequate antifungal treatment guided by susceptibility results. Of note, all candidemia episodes were detected using blood cultures. Molecular methods were not employed for diagnosing candidemia in this study.

### 4.2. Resistance Investigation

With microbroth AFST, resistance was in general rarely observed in this study, especially for azoles and amphotericin B. Previous resistance investigations from Iran also demonstrated limited resistance [13,28], which could be due to the limited administration of antifungals drugs in routine clinical use in Iran. Nonetheless, antifungal resistance is globally increasing, warranting continued surveillance. Among the isolates with reduced susceptibility, one *C. lusitaniae* was resistant to fluconazole. While most strains are susceptible, this species is known to rapidly acquire antifungal resistance, which could result in treatment failure [34]. Furthermore, elevated MICs were observed for some *C. albicans* isolates for echinocandins, with a single isolate resistant for anidulafungin. Although resistance in yeasts against this antifungal class is rarely found, resistant isolates are often from patients who underwent echinocandin treatment, indicating the resistance is possibly therapy-induced [35,36]. In this study none of the patients from whom the *C. albicans* strains with elevated MICs were isolated, were treated with echinocandins. Although transmission of echinocandin-resistant isolates cannot be ruled out, it is rarely reported, making prior colonization with these echinocandin-resistant isolates more likely.

### 4.3. Outbreak Investigation with STR Genotyping

With STR genotyping, multiple clusters were found that frequently consisted of isolates from multiple hospitals. Within these clusters, isolates originated from multiple departments and hospitals, suggesting potential intra-hospital nosocomial transmission. This potential nosocomial transmission needs to be further investigated with a whole genome sequencing (WGS) single nucleotide polymorphism (SNP) analysis. We previously found for other yeast species that STR analyses cannot distinguish isolates differing less than 150 SNPs, which are not clonal but closely related nonetheless [37,38]. For *C. parapsilosis*, numerous nosocomial outbreaks have been reported, frequently caused by fluconazole-resistant strains [12,39,40]. In most reported outbreaks, the source is unknown, but some studies suggest the hands of health care workers or contaminated nosocomial surfaces and equipment [12,41]. Nonetheless, *C. parapsilosis* strains are known to persist despite infection control measures in healthcare settings [42,43]. Recently, transmission of fluconazole-resistant *C. parapsilosis* between two hospitals was reported in Canada, highlighting the need for adequate genomic surveillance [44].

Study limitations include the restriction of genotyping to *C. parapsilosis*, while potential nosocomial transmission of other species is also possible. In addition, to confirm the suspected clonal transmission within the hospitals, WGS analysis would have been required. Furthermore, the current study did not determine whether enforced hand hygiene followed by genotyping of *C. parapsilosis* candidemia episodes is effective to halt clonal transmission within these hospitals and to what extent. Finally, the current study was conducted over a period of two years, which only provides a limited view of the candidemia epidemiology in Iran. For a comprehensive overview, long-term follow-up studies should be conducted to determine whether the species composition further moves from *C. albicans* to NAC yeast species, as this can rapidly shift.

To conclude, the current study demonstrates a high proportion of NAC yeasts causing candidemia in Mashhad, Iran, with a high mortality rate. This emphasis the importance of adequate diagnosis and appropriate antifungal treatment based on AFST data. Additionally, the finding of multiple STR clusters might be caused by nosocomial transmission, which, if confirmed by WGS SNP analysis, would suggest the need for additional infection prevention measures. 

## Figures and Tables

**Table 1:** Patients’ characteristics and epidemiology are divided between multiple *Candida* species. In parentheses, the percentages within each species are displayed.

Characteristic	Overall (*n* = 160, 100%)	*C. parapsilosis* (*n* = 58, 36%)	*C. albicans* (*n* = 52, 33%)	*C. tropicalis* (*n* = 20, 13%)	*C. glabrata* (*n* = 15, 10%)	*C. krusei* (*n* = 7, 4%)	Other Yeasts ^1^ (*n* = 8, 5%)
Age							
<16 years	63 (39)	28 (48)	23 (44)	5 (25)	3 (20)	-	4 (50)
≥16 years	96 (60)	30 (52)	29 (56)	15 (75)	12 (80)	7 (100)	3 (38)
Unknown	1 (1)	-	-	-	-	-	1 (13)
Sex							
Male	83 (52)	28 (48)	33 (63)	7 (35)	8 (53)	4 (57)	3 (38)
Female	77 (48)	30 (52)	19 (37)	13 (65)	7 (47)	3 (43)	5 (63)
Hospital							
Emam Reza	96 (60)	41 (70)	27 (52)	8 (40)	13 (87)	4 (57)	3 (38)
Doctor Sheikh	42 (26)	14 (24)	20 (38)	4 (20)	1 (7)	-	3 (38)
Ghaem	15 (9)	-	5 (10)	5 (25)	-	3 (42)	2 (25)
22 Bahman	5 (3)	2 (3)	-	2 (10)	1 (7)	-	-
Arya	2 (1)	1 (2)	-	1 (5)	-	-	-
Wards							
ICU	79 (49)	25 (43)	30 (58)	6 (30)	9 (60)	6 (86)	3 (38)
Internal	51 (32)	24 (41)	16 (30)	3 (15)	4 (27)	-	4 (50)
Emergency	13 (8)	5 (9)	4 (8)	1 (5)	2 (13)	1 (14)	-
Surgery	5 (3)	3 (5)	1 (2)	1 (5)	-	-	-
Other	12 (8)	1 (2)	1 (2)	9 (45)	-	-	1 (13)
Underlying conditions							
Cardiovascular complications	13 (8)	5 (9)	3 (6)	4 (20)	-	-	1 (14)
Malignancy	25 (16)	8 (14)	10 (19)	4 (20)	2 (13)	1 (14)	-
Diabetes	35 (22)	13 (22)	9 (17)	4 (20)	5 (33)	3 (43)	1 (14)
Internal complications	67 (42)	26 (45)	20 (38)	8 (20)	7 (47)	2 (29)	4 (57)
Cerebrospinal complications	5 (3)	1 (2)	3 (6)	-	-	1 (14)	-
None	14 (9)	5 (9)	7 (13)	-	1 (7)	-	1 (14)
Antifungal treatment							
Fluconazole	48 (30)	10 (17)	16 (31)	3 (15)	12 (80)	3 (43)	4 (50)
Liposomal Amphotericin B	40 (25)	7 (12)	20 (38)	9 (45)	1 (7)	3 (43)	-
Caspofungin	3 (2)	1 (2)	1 (2)	1 (5)	-	-	-
Clotrimazole	3 (2)	-	3 (6)	-	-	-	-
Nystatin	2 (1)	2 (3)	-	-	-	-	
Fluconazole and nystatin	2 (1)	2 (3)	-	-	-	-	-
Fluconazole and amphotericin B	7 (4)	2 (3)	3 (6)	-	-	1 (14)	1 (13)
Fluconazole and caspofungin	1 (1)	-	1 (2)	-	-	-	-
Not treated	54 (34)	34 (59)	8 (15)	7 (35)	2 (13)	-	3 (38)
Outcome							
Died	82 (51)	33 (57)	20 (39)	12 (60)	10 (67)	5 (61)	2 (25)
Survived	74 (46)	25 (43)	31 (60)	8 (40)	5 (33)	1 (14)	4 (50)
Unknown	4 (3)	-	1 (2)	-	-	1 (14)	2 (25)

^1^ Other species comprise six *Candida lusitaniae*, one *Candida dubliniensis*, and one *Meyerozyma guilliermondii*. ICU, intensive care unit.

**Table 2:** In vitro antifungal susceptibility profile (µg/mL) of 131 *Candida* isolates, comprising 48 *C. albicans*, 43 *C. parapsilosis*, 16 *C. tropicalis*, 11 *C. glabrata*, 7 *C. krusei*, and 6 *C. lusitaniae*, according to CLSI M27 guidelines.

Antifungal Drug	Species	Range	GM	MIC_50_	MIC_90_	*n* Resistant/Non-WT (%)
Amphotericin B	*C. albicans*	0.125–4	0.545	0.5	1	1 (2)
	*C. parapsilosis*	0.031–1	0.500	0.5	1	0
	*C. tropicalis*	0.25–1	0.569	0.5	1	0
	*C. glabrata*	0.5–1	0.730	1	1	0
	*C. krusei*	1	1	1	N/A	0
	*C. lusitaniae*	0.5–1	0.630	0.5	N/A	0
Fluconazole	*C. albicans*	0.125–4	0.380	0.25	1	0
	*C. parapsilosis*	0.125–4	0.412	0.5	1	0
	*C. tropicalis*	0.25–1	0.595	1	1	0
	*C. glabrata*	0.25–4	2.416	4	4	0
	*C. krusei*	8–32	17.665	16	N/A	N/A
	*C. lusitaniae*	0.5–2	0.707	0.5	N/A	1 (17)
Voriconazole	*C. albicans*	0.032–0.125	0.036	0.032	0.064	0
	*C. parapsilosis*	0.032–0.125	0.035	0.032	0.064	0
	*C. tropicalis*	0.032–0.125	0.051	0.064	0.125	0
	*C. glabrata*	0.032–0.125	0.050	0.064	0.125	0
	*C. krusei*	0.064–0.25	0.125	0.125	N/A	0
	*C. lusitaniae*	0.032	0.032	0.032	N/A	N/A
Micafungin	*C. albicans*	0.016–0.5	0.022	0.016	0.064	0
	*C. parapsilosis*	0.016–1	0.255	0.5	1	0
	*C. tropicalis*	0.016–0.032	0.017	0.016	0.032	0
	*C. glabrata*	0.016	0.016	0.016	0.016	0
	*C. krusei*	0.064–0.125	0.103	0.125	N/A	0
	*C. lusitaniae*	0.016–0.064	0.029	0.032	N/A	0
Anidulafungin	*C. albicans*	0.016–1	0.027	0.016	0.25	1 (2)
	*C. parapsilosis*	0.016–2	0.376	1	1	0
	*C. tropicalis*	0.016–0.064	0.029	0.032	0.064	0
	*C. glabrata*	0.016–0.064	0.032	0.032	0.064	0
	*C. krusei*	0.032–0.125	0.070	0.064	N/A	0
	*C. lusitaniae*	0.032–0.064	0.057	0.064	N/A	0

GM, geometric mean; MIC, minimum inhibitory concentration; N/A, non-available.

## Data Availability

The original contributions presented in the study are included in the article/Supplementary Material, further inquiries can be directed to the corresponding authors.

## Supplementary Materials

The following supporting information can be downloaded at: https://www.mdpi.com/article/10.3390/jof10070481/s1, Figure S1: UPGMA dendrogram of 57 *C. parapsilosis* isolates; Table S1: In vitro minimum inhibitory concentrations against common antifungals according to CLSI M27 guidelines for *Candida* isolates.
