# Supplementary material for: Epidemiology of Candidemia in Mashhad, Northeast Iran: A Prospective Multicenter Study (2019–2021)
Source: J Fungi (Basel). 2024 Jul 12;10(7):481. doi: 10.3390/jof10070481 (PMC11277834; doi:10.3390/jof10070481)
Supplement: Supplementary file 1 [file jof-10-00481-s001.zip › Figure S1.pptx]

## Slide 1
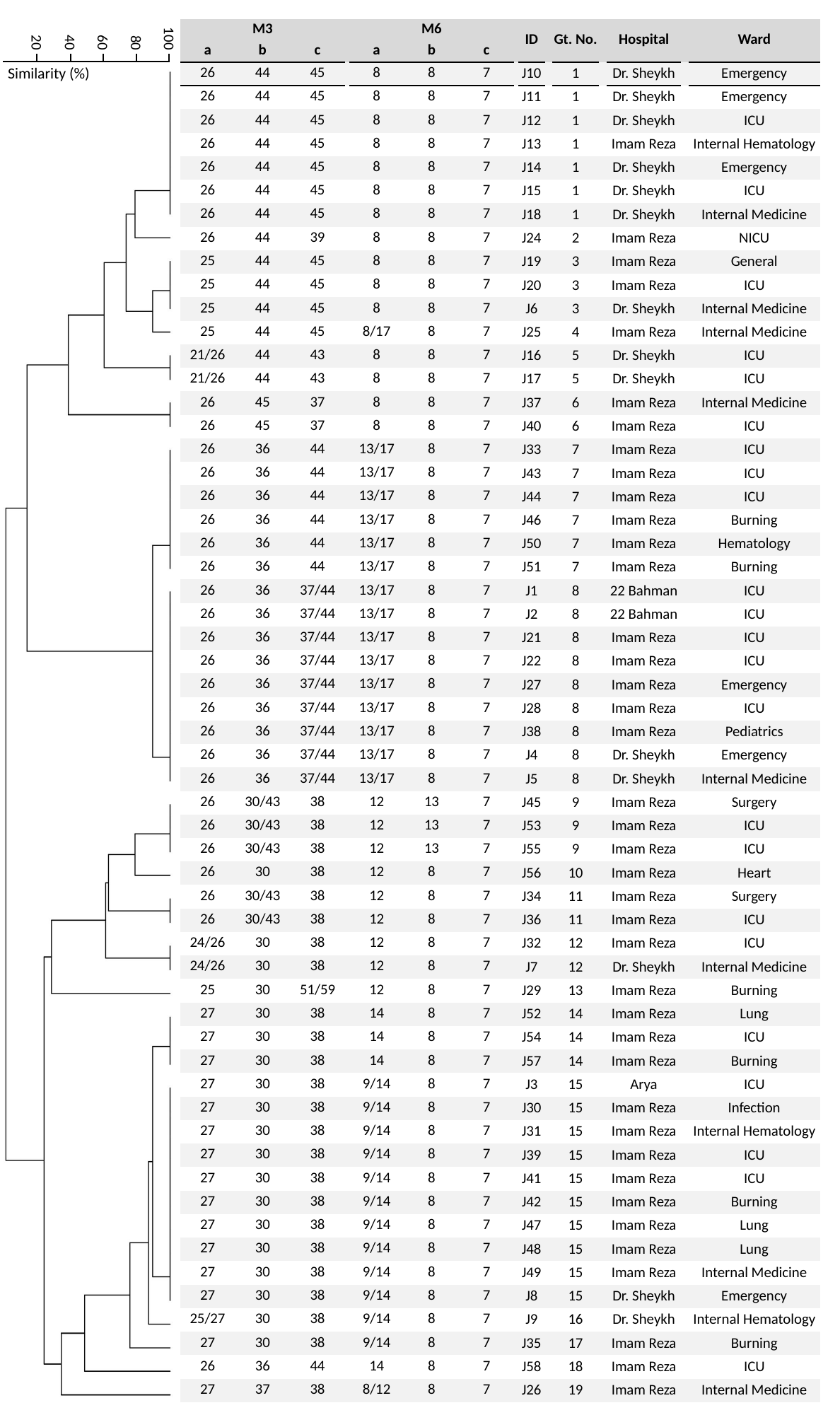

| M3 | | | | M6 | | | | ID | | Gt. No. | | Hospital | | Ward |
| --- | --- | --- | --- | --- | --- | --- | --- | --- | --- | --- | --- | --- | --- | --- |
| a | b | c | | a | b | c | | | | | | | | |
| 26 | 44 | 45 | | 8 | 8 | 7 | | J10 | | 1 | | Dr. Sheykh | | Emergency |
| 26 | 44 | 45 | | 8 | 8 | 7 | | J11 | | 1 | | Dr. Sheykh | | Emergency |
| 26 | 44 | 45 | | 8 | 8 | 7 | | J12 | | 1 | | Dr. Sheykh | | ICU |
| 26 | 44 | 45 | | 8 | 8 | 7 | | J13 | | 1 | | Imam Reza | | Internal Hematology |
| 26 | 44 | 45 | | 8 | 8 | 7 | | J14 | | 1 | | Dr. Sheykh | | Emergency |
| 26 | 44 | 45 | | 8 | 8 | 7 | | J15 | | 1 | | Dr. Sheykh | | ICU |
| 26 | 44 | 45 | | 8 | 8 | 7 | | J18 | | 1 | | Dr. Sheykh | | Internal Medicine |
| 26 | 44 | 39 | | 8 | 8 | 7 | | J24 | | 2 | | Imam Reza | | NICU |
| 25 | 44 | 45 | | 8 | 8 | 7 | | J19 | | 3 | | Imam Reza | | General |
| 25 | 44 | 45 | | 8 | 8 | 7 | | J20 | | 3 | | Imam Reza | | ICU |
| 25 | 44 | 45 | | 8 | 8 | 7 | | J6 | | 3 | | Dr. Sheykh | | Internal Medicine |
| 25 | 44 | 45 | | 8/17 | 8 | 7 | | J25 | | 4 | | Imam Reza | | Internal Medicine |
| 21/26 | 44 | 43 | | 8 | 8 | 7 | | J16 | | 5 | | Dr. Sheykh | | ICU |
| 21/26 | 44 | 43 | | 8 | 8 | 7 | | J17 | | 5 | | Dr. Sheykh | | ICU |
| 26 | 45 | 37 | | 8 | 8 | 7 | | J37 | | 6 | | Imam Reza | | Internal Medicine |
| 26 | 45 | 37 | | 8 | 8 | 7 | | J40 | | 6 | | Imam Reza | | ICU |
| 26 | 36 | 44 | | 13/17 | 8 | 7 | | J33 | | 7 | | Imam Reza | | ICU |
| 26 | 36 | 44 | | 13/17 | 8 | 7 | | J43 | | 7 | | Imam Reza | | ICU |
| 26 | 36 | 44 | | 13/17 | 8 | 7 | | J44 | | 7 | | Imam Reza | | ICU |
| 26 | 36 | 44 | | 13/17 | 8 | 7 | | J46 | | 7 | | Imam Reza | | Burning |
| 26 | 36 | 44 | | 13/17 | 8 | 7 | | J50 | | 7 | | Imam Reza | | Hematology |
| 26 | 36 | 44 | | 13/17 | 8 | 7 | | J51 | | 7 | | Imam Reza | | Burning |
| 26 | 36 | 37/44 | | 13/17 | 8 | 7 | | J1 | | 8 | | 22 Bahman | | ICU |
| 26 | 36 | 37/44 | | 13/17 | 8 | 7 | | J2 | | 8 | | 22 Bahman | | ICU |
| 26 | 36 | 37/44 | | 13/17 | 8 | 7 | | J21 | | 8 | | Imam Reza | | ICU |
| 26 | 36 | 37/44 | | 13/17 | 8 | 7 | | J22 | | 8 | | Imam Reza | | ICU |
| 26 | 36 | 37/44 | | 13/17 | 8 | 7 | | J27 | | 8 | | Imam Reza | | Emergency |
| 26 | 36 | 37/44 | | 13/17 | 8 | 7 | | J28 | | 8 | | Imam Reza | | ICU |
| 26 | 36 | 37/44 | | 13/17 | 8 | 7 | | J38 | | 8 | | Imam Reza | | Pediatrics |
| 26 | 36 | 37/44 | | 13/17 | 8 | 7 | | J4 | | 8 | | Dr. Sheykh | | Emergency |
| 26 | 36 | 37/44 | | 13/17 | 8 | 7 | | J5 | | 8 | | Dr. Sheykh | | Internal Medicine |
| 26 | 30/43 | 38 | | 12 | 13 | 7 | | J45 | | 9 | | Imam Reza | | Surgery |
| 26 | 30/43 | 38 | | 12 | 13 | 7 | | J53 | | 9 | | Imam Reza | | ICU |
| 26 | 30/43 | 38 | | 12 | 13 | 7 | | J55 | | 9 | | Imam Reza | | ICU |
| 26 | 30 | 38 | | 12 | 8 | 7 | | J56 | | 10 | | Imam Reza | | Heart |
| 26 | 30/43 | 38 | | 12 | 8 | 7 | | J34 | | 11 | | Imam Reza | | Surgery |
| 26 | 30/43 | 38 | | 12 | 8 | 7 | | J36 | | 11 | | Imam Reza | | ICU |
| 24/26 | 30 | 38 | | 12 | 8 | 7 | | J32 | | 12 | | Imam Reza | | ICU |
| 24/26 | 30 | 38 | | 12 | 8 | 7 | | J7 | | 12 | | Dr. Sheykh | | Internal Medicine |
| 25 | 30 | 51/59 | | 12 | 8 | 7 | | J29 | | 13 | | Imam Reza | | Burning |
| 27 | 30 | 38 | | 14 | 8 | 7 | | J52 | | 14 | | Imam Reza | | Lung |
| 27 | 30 | 38 | | 14 | 8 | 7 | | J54 | | 14 | | Imam Reza | | ICU |
| 27 | 30 | 38 | | 14 | 8 | 7 | | J57 | | 14 | | Imam Reza | | Burning |
| 27 | 30 | 38 | | 9/14 | 8 | 7 | | J3 | | 15 | | Arya | | ICU |
| 27 | 30 | 38 | | 9/14 | 8 | 7 | | J30 | | 15 | | Imam Reza | | Infection |
| 27 | 30 | 38 | | 9/14 | 8 | 7 | | J31 | | 15 | | Imam Reza | | Internal Hematology |
| 27 | 30 | 38 | | 9/14 | 8 | 7 | | J39 | | 15 | | Imam Reza | | ICU |
| 27 | 30 | 38 | | 9/14 | 8 | 7 | | J41 | | 15 | | Imam Reza | | ICU |
| 27 | 30 | 38 | | 9/14 | 8 | 7 | | J42 | | 15 | | Imam Reza | | Burning |
| 27 | 30 | 38 | | 9/14 | 8 | 7 | | J47 | | 15 | | Imam Reza | | Lung |
| 27 | 30 | 38 | | 9/14 | 8 | 7 | | J48 | | 15 | | Imam Reza | | Lung |
| 27 | 30 | 38 | | 9/14 | 8 | 7 | | J49 | | 15 | | Imam Reza | | Internal Medicine |
| 27 | 30 | 38 | | 9/14 | 8 | 7 | | J8 | | 15 | | Dr. Sheykh | | Emergency |
| 25/27 | 30 | 38 | | 9/14 | 8 | 7 | | J9 | | 16 | | Dr. Sheykh | | Internal Hematology |
| 27 | 30 | 38 | | 9/14 | 8 | 7 | | J35 | | 17 | | Imam Reza | | Burning |
| 26 | 36 | 44 | | 14 | 8 | 7 | | J58 | | 18 | | Imam Reza | | ICU |
| 27 | 37 | 38 | | 8/12 | 8 | 7 | | J26 | | 19 | | Imam Reza | | Internal Medicine |
100
20
40
60
80
Similarity (%)
