# Supplementary material for: Epidemiology of Candidemia in Mashhad, Northeast Iran: A Prospective Multicenter Study (2019–2021)
Source: J Fungi (Basel). 2024 Jul 12;10(7):481. doi: 10.3390/jof10070481 (PMC11277834; doi:10.3390/jof10070481)
Supplement: Supplementary file 1 [file jof-10-00481-s001.zip › Table S1.pdf]

Table S1: ***In vitro*** **minimum inhibitory concentrations against common antifungals according to CLSI M27 guidelines for** ***Candida*** **isolates.** All values are in µg/mL.

ID	Species	AMB	FLU	VOR	MFG	AFG
1	*C. parapsilosis*	0.5	1	0.032	0.5	1
15	*C. tropicalis*	1	0.25	0.032	0.032	0.064
26	*C. tropicalis*	0.5	0.5	0.064	0.016	0.064
39	*C. glabrata*	1	4	0.064	0.016	0.032
53	*C. albicans*	1	2	0.125	0.016	0.064
55	*C. albicans*	1	0.125	0.032	0.016	0.016
56	*C. albicans*	0.5	0.25	0.032	0.016	0.016
57	*C. albicans*	0.5	0.25	0.032	0.016	0.016
59	*C. parapsilosis*	1	1	0.032	0.5	1
60	*C. parapsilosis*	1	1	0.032	0.5	1
61	*C. parapsilosis*	1	1	0.032	0.016	1
62	*C. albicans*	0.5	0.5	0.032	0.016	0.016
63	*C. parapsilosis*	1	0.25	0.032	0.016	1
64	*C. albicans*	0.5	0.5	0.032	0.016	0.016
65	*C. parapsilosis*	1	0.25	0.064	0.5	1
68	*C. tropicalis*	0.5	1	0.064	0.016	0.032
69	*C. tropicalis*	0.5	1	0.064	0.016	0.016
70	*C. albicans*	0.5	1	0.032	0.5	0.5
71	*C. tropicalis*	0.5	1	0.125	0.016	0.064
72	*C. tropicalis*	0.5	1	0.064	0.016	0.064
73	*C. glabrata*	1	2	0.032	0.016	0.064
74	*C. lusitaniae*	1	2	0.032	0.016	0.064
75	*C. albicans*	1	0.5	0.032	0.016	0.125
77	*C. albicans*	1	0.125	0.032	0.016	0.016
78	*C. tropicalis*	1	0.25	0.032	0.016	0.016
80	*C. parapsilosis*	1	0.5	0.032	1	1
81	*C. parapsilosis*	1	0.5	0.032	0.016	0.032
82	*C. parapsilosis*	1	0.5	0.032	1	1
83	*C. parapsilosis*	1	0.5	0.032	1	1
84	*C. parapsilosis*	0.5	0.5	0.032	1	1
85	*C. albicans*	0.5	0.125	0.032	0.5	0.016
86	*C. parapsilosis*	0.5	0.5	0.032	1	1
87	*C. parapsilosis*	0.5	1	0.032	1	1
88	*C. parapsilosis*	0.5	1	0.032	1	1
89	*C. parapsilosis*	1	0.5	0.032	0.5	1
98	*C. tropicalis*	0.5	1	0.064	0.016	0.032
99	*C. krusei*	1	32	0.125	0.125	0.064
100	*C. albicans*	0.5	0.25	0.032	0.016	0.016
102	*C. albicans*	0.5	0.25	0.032	0.016	0.016
103	*C. parapsilosis*	0.032	0.25	0.064	0.016	0.016
104	*C. tropicalis*	0.25	0.25	0.032	0.016	0.016
106	*C. parapsilosis*	0.125	0.25	0.032	0.5	0.5

107	*C. tropicalis*	1	1	0.064	0.016	0.016
109	*C. albicans*	0.5	0.125	0.032	0.016	0.016
110	*C. parapsilosis*	0.5	0.5	0.032	0.5	1
111	*C. tropicalis*	0.5	0.5	0.032	0.016	0.016
112	*C. albicans*	0.5	0.5	0.032	0.016	0.016
113	*C. parapsilosis*	0.5	0.25	0.032	0.016	0.016
114	*C. parapsilosis*	0.5	0.5	0.032	0.25	0.25
115	*C. albicans*	0.5	0.5	0.032	0.016	0.016
116	*C. krusei*	1	32	0.125	0.125	0.125
117	*C. parapsilosis*	1	0.25	0.032	0.016	0.016
118	*C. albicans*	0.5	0.25	0.032	0.016	0.016
120	*C. parapsilosis*	0.5	0.25	0.032	0.5	0.5
121	*C. krusei*	1	32	0.125	0.064	0.064
122	*C. albicans*	0.5	0.125	0.032	0.016	0.016
123	*C. krusei*	1	8	0.064	0.064	0.064
124	*C. parapsilosis*	0.5	0.125	0.032	0.5	1
125	*C. albicans*	0.5	0.125	0.032	0.016	0.016
126	*C. glabrata*	1	2	0.032	0.016	0.032
127	*C. lusitaniae*	1	0.5	0.032	0.016	0.032
128	*C. albicans*	1	1	0.032	0.016	0.016
129	*C. parapsilosis*	0.064	0.5	0.032	0.032	0.032
130	*C. albicans*	0.5	0.5	0.032	0.016	0.16
131	*C. albicans*	0.5	0.25	0.032	0.016	0.016
132	*C. albicans*	0.125	0.25	0.032	0.016	0.016
133	*C. parapsilosis*	0.125	0.125	0.032	0.5	0.5
134	*C. parapsilosis*	0.125	0.25	0.032	1	1
135	*C. parapsilosis*	0.125	0.25	0.032	0.5	0.5
136	*C. albicans*	0.25	0.5	0.032	0.5	0.5
137	*C. parapsilosis*	0.5	0.25	0.032	0.5	1
138	*C. parapsilosis*	0.5	0.125	0.032	0.5	0.5
139	*C. parapsilosis*	0.5	0.125	0.032	0.5	0.5
140	*C. tropicalis*	1	0.5	0.064	0.016	0.032
142	*C. albicans*	0.5	1	0.032	0.016	0.016
143	*C. albicans*	0.5	0.125	0.032	0.016	0.016
144	*C. glabrata*	0.5	4	0.064	0.016	0.032
145	*C. glabrata*	0.5	4	0.064	0.016	0.032
147	*C. tropicalis*	0.5	1	0.032	0.016	0.032
148	*C. parapsilosis*	0.5	0.5	0.032	1	0.5
149	*C. albicans*	0.5	0.25	0.032	0.016	1
150	*C. albicans*	0.5	2	0.032	0.032	0.032
151	*C. albicans*	0.5	0.25	0.032	0.016	0.016
152	*C. albicans*	0.5	0.25	0.032	0.016	0.016
153	*C. albicans*	0.5	1	0.032	0.016	0.016
154	*C. albicans*	0.5	0.5	0.032	0.016	0.016
155	*C. albicans*	0.25	0.25	0.032	0.016	0.016
156	*C. glabrata*	0.5	2	0.032	0.016	0.032

157	*C. glabrata*	0.5	2	0.032	0.016	0.032
158	*C. parapsilosis*	0.5	0.25	0.032	0.5	1
159	*C. albicans*	0.5	0.25	0.064	0.032	0.5
160	*C. albicans*	0.5	0.5	0.064	0.016	0.016
161	*C. lusitaniae*	0.5	1	0.032	0.064	0.064
162	*C. glabrata*	0.5	0.25	0.032	0.016	0.016
163	*C. tropicalis*	0.5	0.25	0.032	0.016	0.032
164	*C. albicans*	0.5	2	0.032	0.016	0.032
165	*C. albicans*	1	1	0.064	0.032	0.064
166	*C. parapsilosis*	1	0.5	0.064	0.016	0.032
167	*C. albicans*	0.5	1	0.032	0.016	0.016
168	*C. lusitaniae*	0.5	0.5	0.032	0.032	0.064
169	*C. parapsilosis*	1	1	0.032	0.125	0.016
170	*C. parapsilosis*	1	4	0.064	0.016	0.032
171	*C. krusei*	1	16	0.125	0.125	0.032
172	*C. glabrata*	1	4	0.064	0.016	0.032
173	*C. krusei*	1	8	0.125	0.125	0.125
174	*C. parapsilosis*	0.5	0.25	0.032	0.25	0.5
175	*C. parapsilosis*	1	0.25	0.032	0.5	0.5
176	*C. parapsilosis*	0.5	0.5	0.032	0.5	1
177	*C. parapsilosis*	1	4	0.125	0.016	0.032
178	*C. lusitaniae*	0.5	0.5	0.032	0.064	0.064
180	*C. lusitaniae*	0.5	0.5	0.032	0.016	0.064
181	*C. albicans*	0.5	0.25	0.032	0.016	0.016
183	*C. parapsilosis*	1	0.25	0.032	1	0.5
184	*C. parapsilosis*	0.25	0.25	0.032	0.5	1
186	*C. tropicalis*	0.25	0.5	0.064	0.016	0.016
187	*C. parapsilosis*	0.125	0.25	0.032	0.5	0.5
188	*C. albicans*	0.125	0.25	0.032	0.016	0.016
189	*C. albicans*	0.5	4	0.064	0.016	0.032
190	*C. albicans*	0.5	0.25	0.032	0.016	0.016
191	*C. albicans*	0.5	0.25	0.064	0.016	0.016
192	*C. albicans*	0.5	0.5	0.125	0.016	0.016
193	*C. parapsilosis*	1	0.25	0.032	1	2
195	*C. tropicalis*	1	1	0.064	0.016	0.032
196	*C. glabrata*	1	4	0.125	0.016	0.032
197	*C. albicans*	1	0.5	0.032	0.016	0.016
198	*C. glabrata*	1	4	0.064	0.016	0.032
199	*C. albicans*	1	0.5	0.032	0.016	0.032
200	*C. albicans*	1	0.25	0.032	0.016	0.016
201	*C. albicans*	4	0.125	0.032	0.016	0.016
203	*C. albicans*	0.5	0.25	0.032	0.25	0.25
206	*C. krusei*	1	16	0.25	0.125	0.064

AMB, amphotericin B; FLU, fluconazole; VOR, Voriconazole; MFG, micafungin; AFG, anidulafungin.
